# Supplementary material for: An Ex-Vivo Culture System of Ovarian Cancer Faithfully Recapitulating the Pathological Features of Primary Tumors
Source: Cells. 2019 Jun 26;8(7):644. doi: 10.3390/cells8070644 (PMC6678777; doi:10.3390/cells8070644)
Supplement: Supplementary file 1 [file cells-08-00644-s001.pdf]

Supplementary: An *Ex-Vivo* Culture System of Ovarian Cancer Faithfully Recapitulating the Pathological Features of Primary Tumors

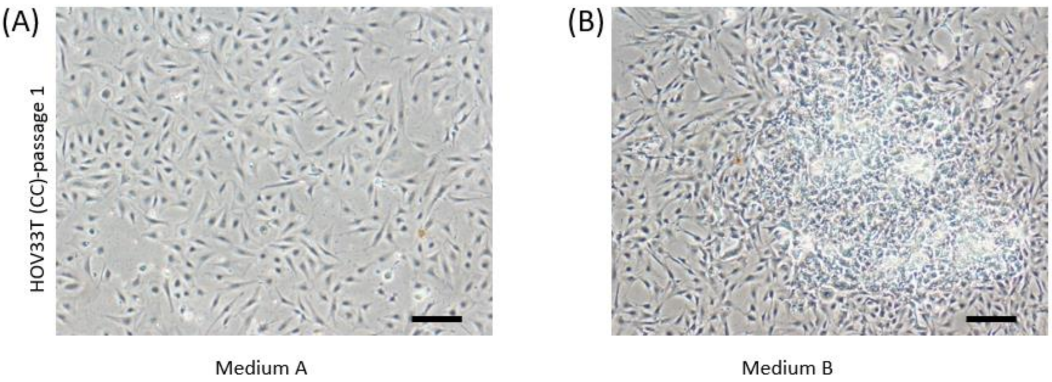

Figure S1. Different proliferation pattern in medium A and B. Representative images of passage 1 HOV33T cells in (A) medium A and (B) Medium B were taken under a phase-contrast microscope. Scale bar 200 μm.

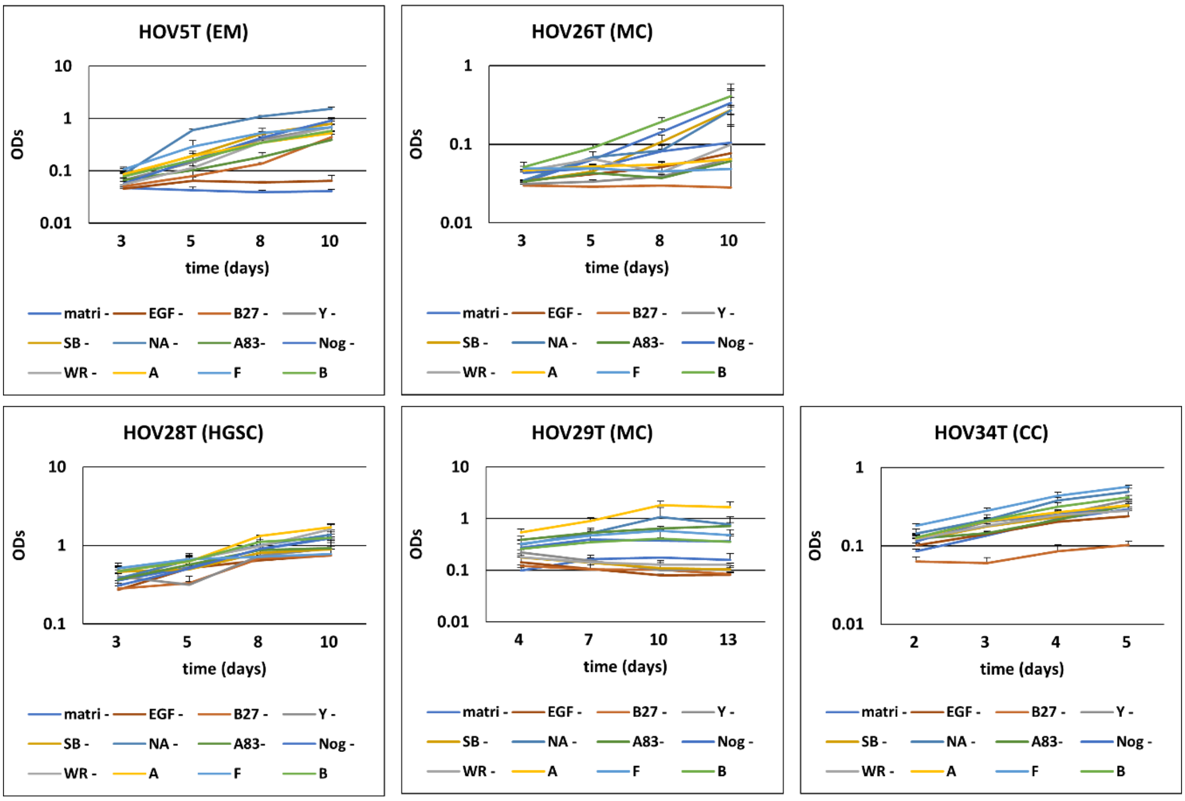

Doubling time (hours) of the cells in each condition

**Cell line**	**HOV5T**	**HOV26T**	**HOV28T**	**HOV29T**	**HOV34T**
**observation period (day)**	**3-8**	**3-8**	**3-8**	**4-10**	**2-5**
**matrigel-coating(-)**	**-430**	**56**	**91**	**175**	**40**
**medium B -EGF**	**299**	**207**	**96**	**-177**	**59**

**medium B -B27**	**83**	**8359**	**94**	**-570**	**101**
**medium B -Y27632**	**46**	**380**	**136**	**-128**	**42**
**medium B -SB202190**	**45**	**72**	**152**	**-209**	**49**
**medium B -Nicotinamide**	**33**	**95**	**86**	**81**	**41**
**medium B -A83-01**	**81**	**805**	**96**	**191**	**51**
**medium B -Noggin**	**48**	**131**	**77**	**315**	**46**
**medium B -Wnt3a/RSPO1**	**41**	**-2237**	**114**	**-304**	**60**
**medium B**	**56**	**63**	**96**	**221**	**37**
**medium A**	**44**	**485**	**80**	**82**	**53**
**F-medium**	**60**	**-1069**	**252**	**172**	**44**

Figure S2. Cell growth curve of HOV lines in different culture conditions. HOV5T (1×10^3 cells/well), HOV26T (1×10^3cells/well), HOV28T (1×10^3cells/well), HOV29T (2×10^3cells/well) and HOV34T (1×10^3cells/well) cells were seeded on matrigel-coated or uncoated (matri-) 96 well plates with the complete medium B. Next day, the complete medium B was replaced with either medium A (A), F-medium, complete medium B (B) or medium B without one component of the supplements, EGF, B27, Y27632 (Y), SB202190 (SB), nicotinamide (NA), A83-01 (A83), Noggin conditioned medium (Nog), or Wnt3A and RSPO1 conditioned medium (WR). Cells were fixed at the indicated time points, and stained by acid red to measure the relative number of cells. The caliculated doubling time in each condition is also presented in the table. Minus value means the cell number rather decreased in the condition. Comparison of individual conditions was summarized in Table 2 as a heatmap.

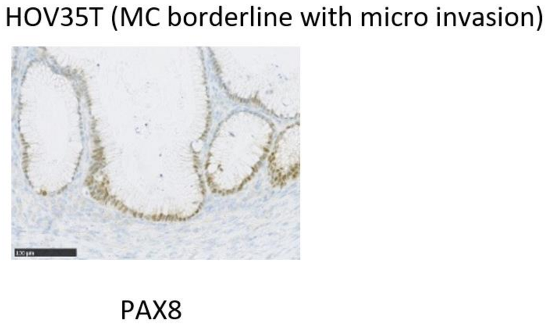

Figure S3. PAX8 immunostaining of original patient tissue of HOV35T. FFPE section of HOV35T original patient tissue was stained for PAX8 as described in Materials and Methods. Scale bar 100 μm.

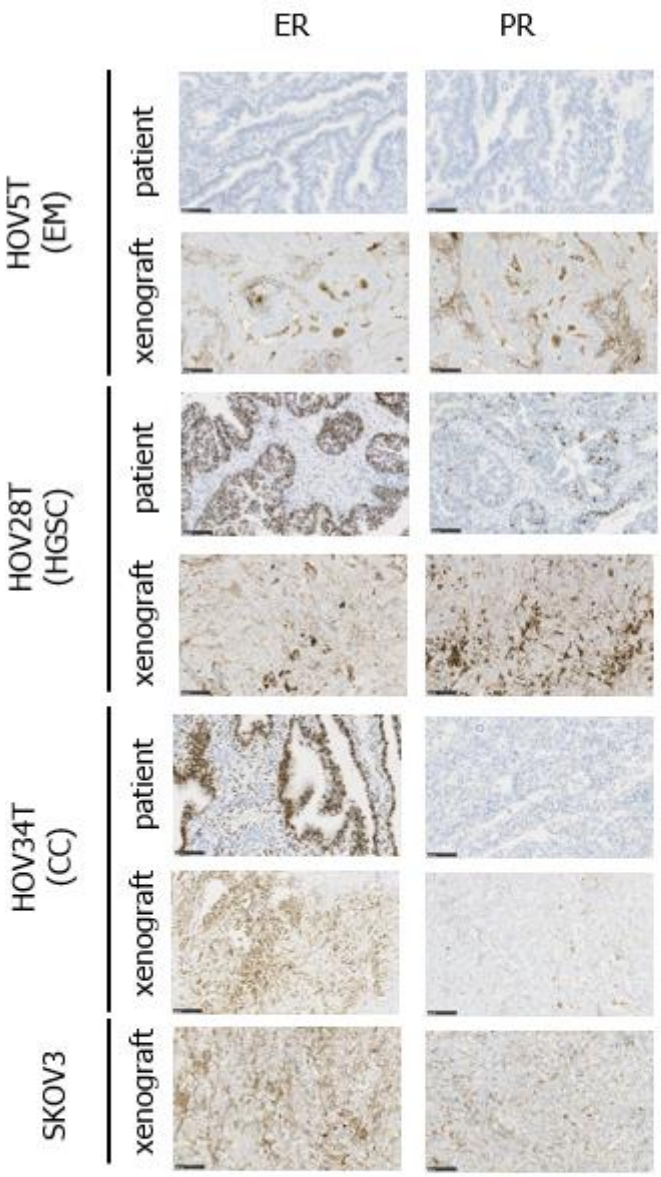

Figure S4. Histotype-specific marker expression fidelity of HOV tumors. FFPE sections of patient tumor tissue, HOV xenograft and SKOV3 xenograft were stained with ER (estrogen receptor) and PR (progesterone receptor). Scale bar 100 μm.

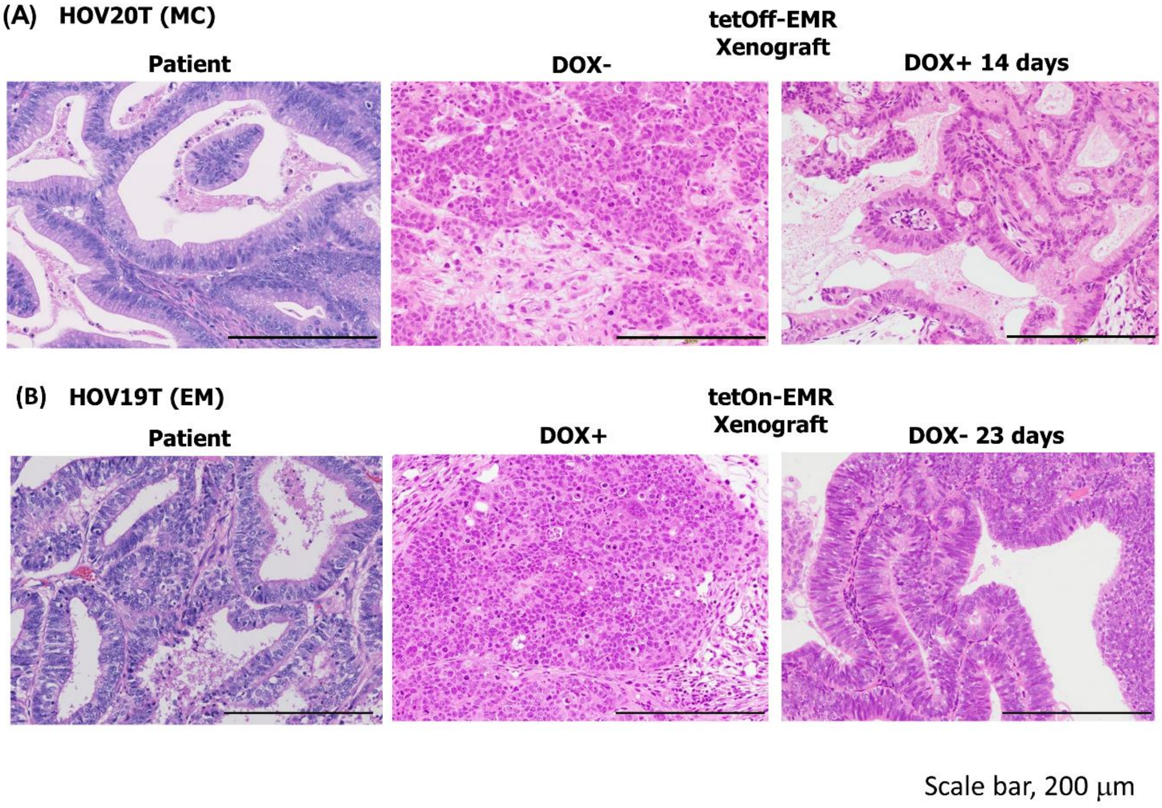

Figure S5. Generation of tumors and recapitulation of original tumor tissues by temporal expression of additional oncogenes in MC and EM type HOV lines. (A) Histopathology of HOV29T/tetOFF/EMR xenograft and original tumor of HOV20T and (B) HOV19T/tetOne-EMR xenograft and original tumor.

Table S1. List of antibodies used for western blotting.

Antibody	Company	Catalog
**ARID1A/BAF250A (D2A8U)**	cell signaling	12354S
**HNF 1B**	Sigma	HPA002083-100U
**PAX8**	Proteintech	10336-1-AP
**Estrogen Receptor α(62A3)**	Cell Signaling	2512
**GAPDH**	Ambion	AM4300
**E-cadherin**	BD Transduction Lab	BD-610181
**Estrogen receptor (ER alfa) (F-10)**	Santa Cruz	sc-8002
**Cytokeratin 7 (RCK105)**	Santa Cruz	sc-23876
**WilmsTumor protein**	abcam	ab89901
**p16INK4A**	BD Pharmingen	550834
**TP53**	Calbiochem (Oncogene Science) Ab6	OP43-100UG
**HPV16 E7(716-325)**	Santa Cruz	sc-51951
**MYC**	Santa Cruz	sc-42
**pan RAS (C-4)**	Santa Cruz	sc-166691
**HPV16 E6 (46A4)**	Home-made, mouse monoclonal, clone 46A4	Raised against N-terminal 16 amino acid peptide of HPV16 E6
**MCM7 (141.2)**	Santa Cruz	sc-9966

Table S2. List of antibodies, antigen retrieval methods and detection systems used for immunohistochemistry.

Antibody	Animal	Company name	Catalog no	Antigen retrival			Detection	
				Solution	Temparature	Time	Name	Company
PAX8	mouse	abcam	ab53490	TRSpH9	98℃	40min	Envision^{TM}Detection System	DAKO
WT1	mouse	DAKO	M3561	TRSpH9	98℃	40min	Envision^{TM}Detection System	DAKO
P16	mouse	BD-Phamingen	550834	Cit.B	121℃	10min	Envision^{TM}Detection System	DAKO
HNF1B	goat	Santa Cruz	sc-7411	TRSpH9	98℃	40min	Simple stain-PO（G）	Nichirei
ARID1A	rabbit	SIGMA	HPA005456	TRSpH9	98℃	40min	Envision^{TM}Detection System	DAKO
CK7	mouse	DAKO	M7018	Cit.B	121℃	10min	Envision^{TM}Detection System	DAKO
E-cadherin	mouse	DAKO	M3612	TRS	121℃	10min	Envision^{TM}Detection System	DAKO
P53	mouse	DAKO	M7001	TRSpH9	98℃	40min	Envision^{TM}Detection System	DAKO
PR	rabbit	Roche	518-102333				ultraViewDAB Universal Kit	Roche
ER	rabbit	Roche	518-107925				ultraViewDAB Universal Kit	Roche
